# Assessment of Multidimensional Health Care Parameters Among Adults in Japan for Developing a Virtual Human Generative Model: Protocol for a Cross-sectional Study

**DOI:** 10.2196/47024

**Published:** 2023-06-09

**Authors:** Masanobu Hibi, Shun Katada, Aya Kawakami, Kotatsu Bito, Mayumi Ohtsuka, Kei Sugitani, Adeline Muliandi, Nami Yamanaka, Takahiro Hasumura, Yasutoshi Ando, Takashi Fushimi, Teruhisa Fujimatsu, Tomoki Akatsu, Sawako Kawano, Ren Kimura, Shigeki Tsuchiya, Yuuki Yamamoto, Mai Haneoka, Ken Kushida, Tomoki Hideshima, Eri Shimizu, Jumpei Suzuki, Aya Kirino, Hisashi Tsujimura, Shun Nakamura, Takashi Sakamoto, Yuki Tazoe, Masayuki Yabuki, Shinobu Nagase, Tamaki Hirano, Reiko Fukuda, Yukari Yamashiro, Yoshinao Nagashima, Nobutoshi Ojima, Motoki Sudo, Naoki Oya, Yoshihiko Minegishi, Koichi Misawa, Nontawat Charoenphakdee, Zhengyan Gao, Kohei Hayashi, Kenta Oono, Yohei Sugawara, Shoichiro Yamaguchi, Takahiro Ono, Hiroshi Maruyama

**Affiliations:** 1 Biological Science Research Kao Corporation Tokyo Japan; 2 Digital Business Creation Kao Corporation Tokyo Japan; 3 Biological Science Research Kao Corporation Tochigi Japan; 4 Analytical Science Research Kao Corporation Tochigi Japan; 5 Sensory Science Research Kao Corporation Tokyo Japan; 6 Hair Care Products Research Kao Corporation Tokyo Japan; 7 Personal Health Care Products Research Kao Corporation Tokyo Japan; 8 Preferred Networks, Inc Tokyo Japan; 9 Ueno-Asagao Clinic Tokyo Japan; 10 Research into Artifacts, Center for Engineering The University of Tokyo Tokyo Japan; 11 Kao Corporation Tokyo Japan

**Keywords:** bacterial profiles, body odor, joint probability distribution model, chiral amino acid, skin surface lipid, multidimensional data, mobile phone

## Abstract

**Background:**

Human health status can be measured on the basis of many different parameters. Statistical relationships among these different health parameters will enable several possible health care applications and an approximation of the current health status of individuals, which will allow for more personalized and preventive health care by informing the potential risks and developing personalized interventions. Furthermore, a better understanding of the modifiable risk factors related to lifestyle, diet, and physical activity will facilitate the design of optimal treatment approaches for individuals.

**Objective:**

This study aims to provide a high-dimensional, cross-sectional data set of comprehensive health care information to construct a combined statistical model as a single joint probability distribution and enable further studies on individual relationships among the multidimensional data obtained.

**Methods:**

In this cross-sectional observational study, data were collected from a population of 1000 adult men and women (aged ≥20 years) matching the age ratio of the typical adult Japanese population. Data include biochemical and metabolic profiles from blood, urine, saliva, and oral glucose tolerance tests; bacterial profiles from feces, facial skin, scalp skin, and saliva; messenger RNA, proteome, and metabolite analyses of facial and scalp skin surface lipids; lifestyle surveys and questionnaires; physical, motor, cognitive, and vascular function analyses; alopecia analysis; and comprehensive analyses of body odor components. Statistical analyses will be performed in 2 modes: one to train a joint probability distribution by combining a commercially available health care data set containing large amounts of relatively low-dimensional data with the cross-sectional data set described in this paper and another to individually investigate the relationships among the variables obtained in this study.

**Results:**

Recruitment for this study started in October 2021 and ended in February 2022, with a total of 997 participants enrolled. The collected data will be used to build a joint probability distribution called a Virtual Human Generative Model. Both the model and the collected data are expected to provide information on the relationships between various health statuses.

**Conclusions:**

As different degrees of health status correlations are expected to differentially affect individual health status, this study will contribute to the development of empirically justified interventions based on the population.

**International Registered Report Identifier (IRRID):**

DERR1-10.2196/47024

## Introduction

### Background

Advances in information technology such as artificial intelligence and the internet of things enable new approaches to human health care [1-5]. Digital data collected through various measurements can be used together to approximate the current health status of humans. Better approximation allows for more personalized and preventive health care by informing the potential risks and developing appropriate interventions and solutions to achieve predictive, preventive, and personalized medicine [6,7]. Elucidating the modifiable risk factors related to lifestyle, diet, and physical activity will help to ensure optimal approaches for individuals in contrast to populations [6,7].

Big data obtained from the results of health examinations or clinical trials may contain a variety of individual laboratory measurements. Using such data, we can train a generative model that reasonably approximates the joint probability distribution. The resulting generative model may contain undiscovered structures among the data and facilitate a better understanding of the data, estimation of missing or corrupt values, detection of outliers, and prediction of unknown data (eg, patient diagnoses) [8-10]. In addition, a generative model can generate synthetic data for various applications, which alleviates privacy concerns when third parties use health care data.

Recent advancements in generative models, particularly those with deep learning, allow the estimation of a joint probability distribution from a high-dimensional data set. We are training this statistical model, hereafter called a Virtual Human Generative Model (VHGM), by combining a commercially available health care data set containing large amounts of relatively low-dimensional data with the high-dimensional, cross-sectional data set described in this paper. In our first incarnation of the VHGM, we incorporated demographics such as age and sex; anthropometrics; biochemical and metabolic profiles of urine, blood, and blood after a glucose tolerance test; vascular, gait, cognitive, and motor functions; RNA, proteins, and metabolites in skin surface lipids (SSLs) [11]; bacterial profiles of the mouth, skin, intestine, scalp; and body odor of the armpit, sole, and scalp. One of the challenges in developing such a high-dimensional statistical model is obtaining the training data set. Deep learning technologies normally require a large amount of data, typically millions or even billions of records. Such data sets for health care measurements, however, are not available. Constructing a new high-dimensional data set with a sufficiently large sample would require prohibitively high financial resources. Therefore, we aim to create a model by combining a large commercially available health care data set (containing >1 million records but relatively low dimensional) with a smaller high-dimensional, cross-sectional data set containing approximately 1000 records. In this study, we describe the details of the protocol for obtaining this cross-sectional data set. Combining these data sets will be discussed in a separate publication.

### Objective

The primary objective of this study is to obtain multidimensional and comprehensive health information to build a data set that will serve as the basis for statistical models such as the VHGM. The secondary objective is to use the same data set to explore the individual relationships among the multidimensional data obtained.

## Methods

### Study Design

**Figure 1:**
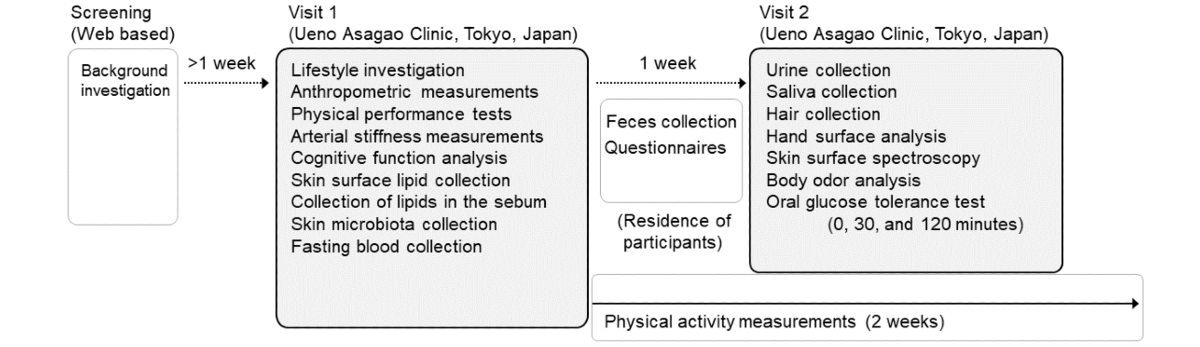
Experimental scheme. Participants were assessed by an initial screening and 2 visits, with a 1-week interval between visits during which they completed a questionnaire and fecal samples were collected. Daily activity was measured continuously over a 2-week period.

A single-center cross-sectional observational study was conducted with adult men and women living in narrowly defined metropolitan areas of Japan (Tokyo, Kanagawa, Chiba, Saitama, Ibaragi, Tochigi, and Gunma prefectures). All measurements were performed by trained research coordinators and medical doctors using standard operating procedures during 2 outpatient visits to the Ueno Asagao Clinic (Tokyo, Japan), 1 week apart (Figure 1). The Strengthening the Reporting of Observational Studies in Epidemiology (STROBE) guidelines were applied according to the study objectives [12].

### Ethics Approval, Informed Consent, and Participation

The study was approved in October 2021 by the institutional review board (IRB) of the Kao Corporation (Tokyo, Japan; approval #K0023-2108) and the Preferred Network, Inc (Tokyo, Japan; approval #ET22110047). Eligibility was evaluated by asking potential participants a few brief questions. After explaining to potential participants that the data obtained in this study may be published after an anonymization process; that statistical models may be created from the obtained data; and that the created statistical models may be provided to external research institutions, commercial companies, and other third parties, they decided whether to participate. All participants provided written informed consent to participate in this study. The consent form explains in detail which data would be used in the study and obtains consent for the use of anonymized data. Participants of this study were provided with digital points worth approximately US $250, which could be used to alleviate any expenses associated with their participation in this study. This trial is registered with the University Hospital Medical Information Network (UMIN; UMIN000045746).

### Participants and Eligibility

Eligible participants were consecutively recruited over a 5-month period from October 2021 to February 2022. The participants were recruited via a website administered by TES Holdings (Tokyo, Japan). We did not perform a formal sample size calculation but set a sample size of 1000 adults to be recruited based on a preliminary calculation that the accuracy of the statistical modeling would not be improved by enrolling >1000 participants. Participants were added and converted to age groups by decade (20-29, 30-39, 40-49, 50-59, 60-69, and >70 years) to evaluate the age of the participants as a numerical value and by sex.

The inclusion criteria were as follows: (1) Japanese men and women aged ≥20 years, (2) individuals who were able to complete the questionnaires and surveys, and (3) individuals who understood the study and could provide written consent. Exclusion criteria were as follows: (1) individuals undergoing hospitalization for serious diseases (diabetes, hypertension, arteriosclerosis, heart disease, malignancy, Alzheimer disease, etc), (2) individuals who could not come to the outpatient unit by themselves, (3) individuals with dementia or suspected dementia, (4) individuals aged ≥60 years who had not received at least 2 doses of the COVID-19 vaccine at least 2 weeks before the visit, (5) individuals who developed symptoms suspicious of COVID-19 infection during the 2 weeks before the visit, (6) individuals who had a pacemaker, (7) individuals who were pregnant or may become pregnant, (8) individuals who had 200 mL of blood drawn within 1 month of the start of this study or >400 mL of blood drawn within 3 months (blood donation, etc) of the start of this study, and (9) individuals whose participation in the study was deemed inappropriate by the study investigators.

### Lifestyle Investigation and Questionnaire

Trained physicians or study coordinators collected information about the participants regarding sex; age; years of education; occupation; medical history; diseases under treatment medication use; smoking habits; alcohol consumption habits; and, for participants aged ≥75 years, nursing care requirements. A 3-day food consumption record for each participant as well as information on hygiene, dental hygiene, and bathing habits; health-related attitudes; skin, scalp, and body odor concerns; hair loss awareness; and, in women, menstrual cycle, were collected. During the study period between visits 1 and 2, participants completed a dietary habits survey using the brief-type self-administered diet history questionnaire [13], the international physical activity questionnaire [14,15], a questionnaire for medical evaluation of old-old [16], a dietary habits questionnaire [17], the Athens insomnia scale [18,19], the Oguri-Shirakawa-Azumi sleep inventory middle-aged and aged version [20], the Berlin questionnaire [21], the World Health Organization—Five Well-Being Index [22], the brief job stress questionnaire [23], the Chalder fatigue scale [24], the Center for Epidemiologic Studies Depression Scale [25,26], the fatigue feelings questionnaire [27], the sun exposure questionnaire [28], the 10-item personality inventory [29,30], the Oxford happiness questionnaire [31], the overactive bladder symptom score [32], the international consultation on incontinence questionnaire–urinary incontinence short form [33], the fecal incontinence quality of life scale [34], the neurogenic bowel dysfunction score [35], the Kupperman menopausal index [36], a modified menstrual distress questionnaire [37,38], climacteric and senescence scores [39], and the Edinburgh postnatal depression scale for those 3 years post partum [40].

### Anthropometric Measurements

Height was measured using a clinical stadiometer. Body weight and body composition, including body fat percentage, body fat mass (kg), lean body mass (kg), total body water (%), and basal metabolic rate (kcal), were measured using a digital bioimpedance scale (InBody 770K, InBody Corp). Visceral fat area and waist circumference were measured using a bioimpedance-type visceral fat meter (EW-FA90; Panasonic Corporation) [41]. Hip circumference was determined using a measuring tape.

### Physical Performance Tests

#### Grip Strength Test and Timed Up-and-Go Test

Grip strength was measured using a Smedley spring-type dynamometer (Grip-D, Takei Scientific Instruments Co, Ltd) [42]. The average value of 2 measurements was recorded. A timed up-and-go test was administered to measure the time required to stand up, walk 3 m, turn, walk back to the chair, and sit down [43].

#### In-Laboratory Gait Parameter Measurement

In-laboratory gait parameters and foot pressure patterns were determined as described previously [44]. Gait speed was measured using a sheet-type pressure sensor (Anima Corporation) placed at the center of a 6.4-m walkway. The measured gait parameters included the following: general parameters—speed (cm/s) and cadence (steps/min); spatial parameters—step length (%), step width (%), gait angle (degree), and toe angle (degree); and temporal duration parameters—double support phase (%), swing phase (%), and stance phase (%) [45]. An average measurement of the 4 trials was obtained for each participant. The foot pressure patterns were measured simultaneously using the sheet-type pressure sensor. The gait parameters and foot pressure on the left and right feet were recorded bilaterally.

#### Physical Activity Measurement

Daily physical activity, gait speed, and number of steps were monitored using a triaxial accelerometer (HW-100, Kao Corporation) that allowed for 14 days of continuous recording, as previously reported [44]. Effective days were defined as the days when the accelerometer was worn for at least 10 hours per day and for at least 7 days [46,47]. The participants used the walking speed measurement app (Chami, InfoDeliver Co Ltd) on their smartphones for 14 consecutive days. The smartphone app that measures walking speed uses a step counter and GPS [48,49]. Walking speed data, including the time of measurement, number of steps, and distance walked, were collected on a dedicated server.

### Blood Pressure and Arterial Stiffness Measurements

Heart rate, systolic blood pressure, and diastolic blood pressure were measured with the participants in a sitting position using a VS-2500 Vascular Screening System (Fukuda Denshi Co, Ltd). Measurements of the cardio-ankle vascular index and ankle brachial index were obtained while the participants assumed a supine position using the VS-2500 Vascular Screening System with an electrocardiogram, phonocardiogram, and brachial and ankle artery pressures and waveforms [50]. The cardio-ankle vascular index was automatically calculated for the left and right sides, and both values were used for analysis.

### Blood, Urine, Saliva, and Hair Collection

Blood samples were obtained from a peripheral vein after overnight fasting or glucose loading. Serum samples were obtained by centrifuging (1500 × g, 15-24 °C, 10 min) blood allowed to stand at room temperature (18-25 °C) for 15 minutes. Plasma samples were obtained by centrifuging (1500 × g, 4 °C, 10 min) blood samples collected in tubes containing EDTA-disodium salt dihydrate or sodium fluoride (glucose). Plasma samples obtained during the oral glucose tolerance test were distributed into tubes containing EDTA-dipotassium salt dihydrate and dipeptidyl peptidase IV inhibitors or sodium fluoride (glucose). Blood samples for ammonia measurements were collected in tubes containing a deproteinizing reagent. Freeze-thaw was performed only once between the collection of biological samples, and all plasma and serum samples were stored at −80 °C until analysis.

Urine samples (4-6 mL) were collected from participants in the morning after overnight fasting for at least 12 hours. All urine samples were stored at −20 °C until laboratory analysis.

Saliva samples were extracted using a standard protocol [51,52]. Saliva samples were obtained from participants seated in a quiet room in the morning between 08:30 AM and 11:30 AM. Participants were asked to accumulate saliva in their mouths and spit into a sterile plastic tube every minute for 20 minutes. The saliva volumes (gram) were weighed in the tube after sample collection [53]. The saliva secretion rate (mL/min) was calculated by dividing the amount of saliva collected by the collection time (20 min). Whole saliva samples were centrifuged at 14,970 × g for 10 minutes at 4 °C, and the supernatant and pellet were stored at −80 °C until analysis. To measure salivary nitrate and nitrite concentrations, the salivary supernatants were mixed with an equal volume of methanol and centrifuged (21,600 × g, 4 °C, 15 min). The supernatants were analyzed using high-performance liquid chromatography (HPLC; ENO-30, Eicom Corp).

All hair samples were collected from the occipital scalp area of each participant. Hair samples were obtained from the edge of each participant’s scalp. Hair thickness was measured with a thickness gauge near the root tip for 10 randomly selected hair fibers from each participant (type 547-401; Mitutoyo Corp). The measured thickness usually corresponds to a small diameter when the cross-sectional shape of the fiber is oval. The curvature of the hair fiber was measured by image analysis of a 2D image of each hair fiber [54].

### Laboratory Analysis

Hematologic parameters, such as white blood cells, red blood cells, hemoglobin level, hematocrit level, platelet cells, mean corpuscular volume, and mean corpuscular hemoglobin, were measured in whole blood samples using standardized methods. The following blood biochemistries and blood electrolytes were measured using standardized methods according to the standard operating procedures of the Health Sciences Research Institute, Inc (Kanagawa, Japan): sodium, potassium, chlorine, calcium, inorganic phosphorus, iron, magnesium, total protein, albumin, albumin:globulin ratio, total bilirubin, aspartate aminotransferase, alanine aminotransferase, γ-glutamyl transpeptidase, alkaline phosphatase, lactate dehydrogenase, creatine phosphokinase, uric acid, urea nitrogen, creatinine, serum triglycerides, total cholesterol, high-density lipoprotein cholesterol, low-density lipoprotein cholesterol, glucose (plasma), hemoglobin A_1c_, ammonia, high-sensitivity C-reactive protein, cortisol, triiodothyronine, thyroxine, dehydroepiandrosterone, testosterone, estradiol, progesterone, follicle-stimulating hormone, luteinizing hormone, prolactin, insulin, free fatty acids, total ketones, acetoacetic acid, and 3-hydroxybutyric acid. Plasma inflammation marker levels were measured by Kao Corporation using a Bio-Plex Pro Human Cytokine Screening Test Kit (Bio-Rad Laboratories Inc, Hercules). The following 27 cytokines were quantified: basic fibroblast growth factor, eotaxin, granulocyte colony-stimulating factor, granulocyte-macrophage colony-stimulating factor, interferon-γ, interleukin (IL)-1β, IL-1ra, IL-2, IL-4, IL-5, IL-6, IL-7, IL-8, IL-9, IL-10, IL-12, IL-13, IL-15, IL-17, interferon-γ–induced protein 10, monocyte chemoattractant protein-1, macrophage inflammatory protein-1α, macrophage inflammatory protein-1 β, platelet-derived growth factor, regulated upon activation in normal T cell expressed and presumably secreted, tumor necrosis factor-, and vascular endothelial growth factor.

Glucose, protein, bilirubin, urobilinogen, ketone bodies, creatinine, occult blood reaction, pH, and gravity were measured in the urinary samples.

Salivary oxytocin concentrations were quantified using an enzyme-linked immunosorbent assay kit (Enzo Life Sciences, Plymouth Meeting). Salivary oxytocin concentrations were evaluated in duplicate and determined using the Epoch2 microplate reader (BioTek Instruments, Inc). Bovine serum albumin (Bio-Rad Laboratories, Inc) was used as a standard to calculate the salivary total protein concentration. The salivary oxytocin concentration was normalized by the salivary total protein concentration in each sample (pg/mg protein), as previously described [55].

### Oral Glucose Tolerance Test

Other than the participants diagnosed with diabetes or other potentially serious illnesses, all participants underwent an oral glucose tolerance test at visit 2 after fasting overnight. After the fasting blood sample was collected, a 75-gram glucose load was ingested. Blood samples were obtained at 30 minutes and 120 minutes after the glucose load. Glucagon, active glucagon-like peptide-1, total glucose-dependent insulinotropic peptide from plasma, insulin, growth hormone, and insulin-like growth factor 1 from the serum were measured using enzyme-linked immunosorbent assay kits (glucagon: Mercodia; active glucagon-like peptide-1: Immune Biology Laboratories; total glucose-dependent insulinotropic peptide: Merck KGaA; and growth hormone and insulin-like growth factor 1: R&D Systems). Plasma glucose and serum insulin were assessed according to standard procedures at the laboratory at Health Sciences Research Institute, Inc (Kanagawa, Japan).

### Liquid Chromatography–Tandem Mass Spectrometry Analysis

#### Chiral Amino Acid Analysis

According to a previous report [56], human plasma was mixed with an aqueous methanol solution and centrifuged at 2130 × g for 10 minutes at 4 °C to remove proteins. After derivatization using 6-aminoquinolyl-N-hydroxysccinimidyl carbamate, the reaction solution was subjected to chiral tandem liquid chromatography–tandem mass spectrometry (LC-MS/MS).

#### Lipid Mediator Analysis

Quantification of lipid mediators was performed using LC-MS/MS, as previously described [57,58]. The plasma was mixed with methanol and centrifuged (20,000 × g, 10 minutes, 4 °C), and the solid-phase extraction was carried out using an Oasis hydrophilic-lipophilic balance solid-phase extraction cartridge (Waters Corporation). Lipid mediators were analyzed using an UltiMate3000 liquid chromatography (LC) system (Thermo Fisher Scientific) coupled with a QTRAP5500 triple-quadrupole mass spectrometer (AB Sciex Pte Ltd) equipped with an electrospray ionization source.

#### Vitamin D Metabolites Analysis

The plasma samples were loaded into Chem Elut S (Agilent Technologies), held for 10 minutes at room temperature and eluted with ethyl acetate. The supernatant was evaporated to complete dryness under a nitrogen stream, and *N*-methyl-1,2,4-triazolinedione solution was added. The reaction solution was subjected to LC-MS/MS. Vitamin D metabolites were analyzed using a QTRAP6500 triple-quadrupole mass spectrometer (AB Sciex Pte Ltd) equipped with a Nexera X2 LC system (Shimadzu). For the quantification of vitamin D metabolites, each peak area of the species was standardized by the peak area of each internal standard.

#### Polyamine Analysis

Plasma samples were mixed with an internal standard solution containing putrescine-d6, spermine-d20 (C/D/N Isotopes), and spermidine-d8 (Toronto Research Chemicals Inc). Acetonitrile (Kanto Chemical Co, Inc) was added to the plasma, and the precipitate was removed by centrifugation at 20,400 × g for 10 minutes at 4 °C. Amino Tag Wako Borate Buffer, 3-aminopyridyl-N-hydroxysuccinimidyl carbamate (APDS) reagent, and APDS Tag Wako Eluent Buffer (Fujifilm Wako Pure Chemical Corporation) were added to the supernatant. Mass spectrometric analysis of the resulting samples was performed by LC-MS/MS (selected reaction monitoring) using an LC system (ACQUITY Premier System, Waters Corporation) coupled with a Xevo tandem triple-quadrupole-mass spectrometry (MS; Waters Corporation) equipped with a heated electrospray source. For quantification of polyamines, the peak areas of putrescine, spermidine, and spermine were standardized by the peak area of each internal standard.

### Hair Loss Determination

Male-pattern and female-pattern hair loss were determined according to the Japanese Dermatological Association’s Guidelines for the diagnosis and treatment of male- and female-pattern hair loss, 2017 version [59]. Male-pattern hair loss was classified by combining the classification method by Norwood [60] and the type II vertex in the classification method by Takashima et al [61], with the type II vertex further divided into 2 groups according to the severity of hair loss. Photographs obtained with a Nikon D5600 from the top of the man’s head and hairline observed from the side were used to classify male-pattern hair loss.

To classify female-pattern hair loss, women’s thinning hair was determined using a total score (0-20 points) of hair appearance (0-10 points), vellus hair rate (0-5 points), hair shedding (0-2 points), and single hair follicular unit (FU; 0-3 points) [62]. A photograph of the midline of the top of the woman’s head was obtained. Using photographs of midline partings, a 5-point scale based on the hair density scale by Sinclair was given a score of 0 to 10 and used to determine thinning of hair in women. An enlarged photograph (20× magnification) of the midline connecting the ears was taken with a digital microscope (KH-7700, Hyrox Co, Ltd), and the numbers of black hairs and gray hairs in a 0.21-cm^2^ area and hair diameter were measured using Image-Pro Plus (ver6.1, Hakuto Co, Ltd). Scores of 0 (vellus hair rate <20%) and 5 (vellus hair rate ≥20%) were used to determine thinning of hair in women. To apply these measurements to determine the initial thinning of hair in women, hair shed during washing was collected with a net (draining net stocking type shallow type for drain holes), and the amount of hair shed (from 10 to 750 hairs) was self-evaluated in 6 stages. The number of hairs lost was divided by the time since the previous hair wash, and the amount of hair shedding was determined. Shedding of 10 to 200 hairs was scored as 0, and hair shedding of ≥400 hairs was scored as 2. The ratio of single hair FUs in the 0.21-cm^2^ area was measured from a 20× magnification image. Single hair FU ratios were scored for women as follows: <25%, score of 0; ≥25% and <50%, score of 1; ≥50% and <75%, score of 2; and ≥75%, score of 3.

### Hand Surface Analysis

Participants were instructed to wash their hands with a commercially available soap [63]. The amount of lactic acid was measured by placing an acrylic cylinder with an inner diameter of 1.5 cm on the hand, adding pure water, and pipetting the mixture. The concentration of l-lactic acid was measured using Lactate Pro 2 (ARKRAY Co Ltd, Inc) with the extracted solution [64]. The measurements were performed twice, and the values were averaged. The skin pH was measured using a multiskin measuring instrument and a skin pH meter (pH 905; Courage+Khazaka Electric GmbH). The surface temperature of the hand was measured using a noncontact thermometer (Shenzhen Hezhizhou Technology Co, Ltd) [65].

### Lipids in the Stratum Corneum and Sebum Analysis

Stratum corneum from the forehead were obtained by stripping with adhesive acrylic film (No. 465 #40, Teraoka Seisakusho). According to a previous report [66], ceramides, free fatty acids, cholesterol, and cholesterol sulfate profiles of the stratum corneum samples were determined using reverse-phase LC-MS.

Face sebum was collected from the forehead using 2 pieces of cigarette paper (Blue double, Rizla Ltd). The sebum was extracted using chloroform or methanol for MS analysis. Sebum MS analysis was performed using flow injection analysis coupled with MS with an ultrahigh performance LC system (Vanquish, Thermo Fisher Scientific) containing a Q-Exactive Focus (Thermo Fisher Scientific) equipped with a heated electrospray source. The Fourier transform MS full-scan mode was used.

### Body Odor Analysis

Participants were instructed not to use products such as deodorants or antiperspirants on their armpits, scalp, and soles of the feet during the night before collection of the body odor sample. Body odor was analyzed using solid-phase microextraction–gas chromatography–MS (SPME-GC-MS). Scalp odor samples were collected by pressing an oil-blotting film (3M Japan) against the parietal scalp ≤4 times at 6 different places and were then stored in a glass vial, immediately frozen, and stored at −80 °C until analysis. Each scalp odor sample was transferred to a 20-mL clear glass vial with a silicone or polytetrafluoroethylene crimp cap for SPME-GC-MS analysis. To collect samples for axillary odor analysis, a cotton pad (Katte Gauze, Ryugu Co, Ltd) infiltrated with 1.2 mL of distilled water was used to swab a 10-cm^2^ area from the armpit at least 10 times for each participant. Foot odor samples were collected from the soles of the feet in the same manner. Each swabbing pad was stored in an aluminum bag with a zipper, immediately frozen, and stored at −80 °C until analysis. Each swabbing pad was centrifuged (6000 revolutions/min, 10 min), and the supernatants were acidified (pH 5.0) with 0.01 N hydrogen chloride and transferred to a 10-mL clear glass vial with a silicone or polytetrafluoroethylene crimp cap for SPME-GC-MS analysis.

### Skin Surface Spectroscopy

#### Advanced Glycation End Product Analysis

The accumulation levels of advanced glycation end products (AGEs) in the skin were measured using an AGE reader (DiagnOptics Technologies) according to the specific protein modifications and fluorescent properties of AGEs [67].

#### Laser Speckle Blood Flowmetry

Facial skin blood flow was measured using laser speckle flowgraphy (a prototype based on LSFG-ANW, Softcare Co, Ltd) [68]. An industrial digital camera (DFK0818UP1300, The Imaging Source GmbH) with a lens (VS-1300VM, VS Technology Corporation) was used to record a series of moving images at a rate of 30 frames/s.

### Cognitive Function Analysis

Cognitive function was assessed using the computer-administered neurocognitive test battery Cognitrax (CNS Vital Signs), for which the validity and reliability have been confirmed [69]. Cognitrax comprises the following battery of tests: a verbal memory test evaluating word learning, word memory, and word recognition, as well as immediate and delayed recall; visual memory test evaluating shape learning ability, shape memory, and shape recognition, as well as immediate and delayed recall; finger-tapping test evaluating motor speed and fine motor control; symbol-digit test evaluating complex information processing accuracy, complex attention, visual perceptual speed, and information processing speed; stroop test evaluating simple reaction time, complex reaction time, stroop reaction time, inhibition and disinhibition, and frontal or executive skills; shifting attention test evaluating executive function, shifting sets (rules, categories, and rapid decision-making), and reaction time; and continuous performance test evaluating the ability to maintain sustained attention, choice reaction time, and impulsivity. A composite neurocognitive index and cognitive domain scores were generated on the basis of the 7 tests.

### Microbiota Analysis

Intestinal microbiota analyses were performed as previously reported [70,71]. Briefly, the participants were instructed to use an FS-0002 tube kit (TechnoSuruga Laboratory Co Ltd) containing guanidine thiocyanate solution to collect fecal samples and store them in a refrigerator (≤4 °C) between visits. Zirconia beads were added to the samples, milled at 5 m/s for 2 minutes using a FastPrep 24 Instrument (MP Biomedicals), and centrifuged for 1 minute at 2350 × g. Bacteria were identified on the basis of the 16S recombinant DNA V3-V4 region. Fragments of the polymerase chain reaction (PCR) mixtures were purified by electrophoresis on 1% agarose gels, followed by PCR Cleanup Filter Plates (Merck Millipore). Illumina paired-end sequencing was performed using the 2 × 300 cycle paired-end method and the MiSeq system (Illumina).

Skin microbiota were collected from the scalp and cheek. To collect scalp microbiota, the hair was parted with a rubber-gloved hand to expose the frontal area of the scalp, and to collect facial skin microbiota, a custom-made sampling template was used to allow for consistent sampling of 4 cm^2^ of the cheek areas. A sterile swab (Nippon Becton Dickinson Company, Ltd) soaked in BBL-prepared saline solution (Becton, Dickinson and Company) was rubbed onto the scalp surface or the designated cheek areas. The head of the swab was cut and placed into a microtube. All samples were frozen at −80 °C until use. The V1-V2 hypervariable region was used as the target for 16S ribosomal RNA (rRNA) gene sequencing. DNA extraction, DNA library preparation, and DNA sequencing were performed by BIKEN Biomics Inc using their standard procedures.

To analyze the saliva microbiota, part of a whole saliva pellet resuspended in sterile water was collected in a 1.5-mL tube and centrifuged for 10 minutes at 15,000 × g at 4 °C. After removing the supernatant, the pellet was stored at −80 °C until the samples were analyzed by Genome Lead Co, Inc. DNA was extracted from each sample using both enzyme treatment and the beads-beating methods (Promega). Then, DNA was extracted from the samples using zirconia beads (EZ-Beads; Promega) and Genfind v2 (Beckman Coulter). The composition of the saliva microbiota was assessed by high-throughput sequencing of the 16S rRNA gene of the V1-V2 region using the Illumina NovaSeq Platform (Illumina). The V1-V2 regions of the 16S rRNA genes from each sample were amplified using primers containing 1 random base sequence at the N position. The 2× KAPA HiFi HotStart ReadyMix (KAPA Biosystems) was used for amplicon PCR and index PCR. Both the PCR products were purified using AMPure XP beads (Beckman Coulter). The amplicon PCR products were purified using AMPure XP beads (Beckman Coulter). NovaSeq sequencing was performed using the NovaSeq 6000 Reagent Kit v1.5 (500 cycles; Illumina) using a 251-bp paired-end sequencing protocol. All 16S rRNA raw sequence data were analyzed using the open-source QIIME2 platform [72], version 2021.2 (qiime2-2021.2).

### SSL‑RNA Analysis

The SSLs collection and SSL-RNA purification were performed as described previously [11]. In brief, an oil-blotting film (3M Japan) was used to wipe the forehead, cheeks, face line, nose, and chin or parietal scalp and stored in a glass vial at −80 °C until analysis. The oil-blotting film was cut into small pieces and homogenized in 1.45 mL of QIAzol (QIAGEN). The SSLs were extracted by placing the supernatant in a tube, adding chloroform, vortexing the mixture for 10 seconds, centrifuging the mixture at 12,000 × g for 15 minutes at 4 °C, and transferring the upper phase to a fresh tube. A RNeasy Mini Kit and QIAcube (QIAGEN) with concomitant DNase I treatment were used to purify the RNA, following the manufacturer’s instructions.

Sequence library preparation was performed using the Ion AmpliSeq Transcriptome Human Gene Expression Kit (Thermo Fisher Scientific), following our previously described modified protocols [11,73,74]. Briefly, the RNA solution was combined with VILO Reaction Mix, SuperScript III enzyme, and T4GP32 (New England Biolabs). The target DNA was amplified using cDNA solution, Ion AmpliSeq HiFi Mix, T4GP32, and the Ion AmpliSeq Transcriptome Human Gene Expression Core Panel with incubation for 20 cycles. We purified the amplified DNA library by binding it to AMPure XP beads (Beckman Coulter), according to the manufacturer’s protocol. A High-Sensitivity D1000 ScreenTape on an Agilent 4200 TapeStation was used to assess the quality of the DNA library. After confirming the quality of the DNA library, the purified library solution was combined with the Ion AmpliSeq HiFi Mix, Ion AmpliSeq Transcriptome Human Gene Expression Core Panel, and VILO Reaction Mix to produce the reaction solution. The adapter sequence was ligated to the library by adding Switch solution, Ion Xpress barcode adapters, and DNA ligase to the reaction solution and then incubating the mixture. The libraries were eluted from the beads using Library Amp Mix (Thermo Fisher Scientific) containing Library Amp Primers. An aliquot of the PCR product was purified using AMPure XP beads, and the supernatant was transferred to fresh PCR tubes. The quality of the sequence library was assessed using the High-Sensitivity D1000 ScreenTape on the Agilent 4200 TapeStation. An Ion Library TaqMan Quantitation Kit (Thermo Fisher Scientific) was used to quantify the library. After loading the DNA library in the Ion Chef System (Thermo Fisher Scientific), the template was prepared and the chip was loaded, and the Ion S5 XL System (Thermo Fisher Scientific) was used to perform the RNA sequencing.

### SSL-Protein Analysis

Proteins for SSL-protein analysis were prepared using S-trap micro columns (ProtiFi), according to the partially modified procedure described in the manufacturer’s protocol. In this study, sodium deoxycholate and sodium lauryl sulfate were used for the protein solubilization solution. Following the removal of SSL-RNA from the upper layer, 2-propanol was added to the residual solution. After centrifuging at 14,000 × g for 5 minutes, the precipitate was dissolved in a protein solubilization solution and applied to an S-trap column. The eluted tryptic digest was concentrated under reduced pressure and desalted using styrene divinylbenzene polymer GL-Tip (GL Sciences) according to the manufacturer’s protocol. The peptide mixtures were subjected to LC-MS/MS. LC separations were performed with a nano-HPLC system Ultimate 3000 (Thermo Fisher Scientific) using an Acclaim PepMap 100 analytical column (Thermo Fisher Scientific). MS detection was performed with a Q-Exactive Plus (Thermo Fisher Scientific) in positive-ion mode and operated in the data-dependent acquisition mode and data-independent acquisition mode. Analysis of the acquired data was performed using Mascot database search (Matrix Science) and DIA-NN software (version 8.1; GitHub) [75].

### SSL-Metabolite Analysis

The SSLs for metabolite analysis were collected by wiping oil-blotting paper around the neck below the face line, and the samples were stored in glass vials at −80 °C until use. To extract metabolites, the oil-blotting film was cut into small pieces and placed in a glass vial with methanol. The supernatant was filtered through a DISMIC-13HP 0.45-μm syringe filter (Advantec) and concentrated under reduced pressure until dry. Each sample was redissolved in 80% (volume/volume) methanol and filtered through a DISMIC-13HP 0.45-μm syringe filter. A portion of the filtrate from each sample was mixed in equal volumes to create a pooled quality control sample.

SSL-metabolite analysis was conducted using an ExionLC system (AB Sciex) coupled to an AB SCIEX X500R electrospray-ionization quadrupole time-of-flight high-resolution MS (AB Sciex). The LC system comprises a 2-channel pump, a degasser, a column oven, an autosampler, and a controller. LC was performed by injecting the sample onto an L-column3 C18 reversed-phase column (Chemicals Evaluation and Research Institute).

We used the sequential window acquisition of all theoretical mass spectra. In total, LC-MS analysis was performed in 10 batches. The quality control dilution series was used to confirm the linearity of the metabolites. Data processing, including automatic peak finding, locally weighted scatterplot smoothing normalization, and identification via spectra matching, was performed using MS-DIAL (version 4.7; downloaded from the PRIMe website) [76]. All automatically detected peaks were checked manually, and the linearity of each metabolite was checked using diluted quality controls, excluding peaks with low linearity (*r*≤0.85).

### Statistical Analysis

#### Data Management

The data of each participant were managed using an electronic database and case record forms. There were 2 levels of data quality control. The first level of quality control was conducted by the investigators while entering the records into the case record form. For the second level, an independent group conducted data monitoring and validation. An electronic database served as a double-entry system to further ensure accurate data input.

#### VHGM Analysis

We will construct a generative model to capture the joint distribution among the aforementioned health care attributes. As the attributes comprise both numerical and categorical values, a model that can handle this type of heterogeneity must be selected. To this end, we will use the heterogeneous incomplete variational autoencoder (HIVAE) [8]. The HIVAE is an extension of a variational autoencoder [77] in which a different likelihood function can be selected depending on the attribute type. For example, we will input the normal distribution for weight and height and the categorical distribution for sex (male, female, and other). Given a set of observed attributes as inputs, we can use the trained model to estimate the parameters of the (conditional) probability distribution of each attribute, including the missing ones. The objective function of HIVAE is called the evidence lower bound, which measures the discrepancy between the training data set and the model. We will separate the entire data set into training and testing sets and train the model to minimize the evidence lower bound to fit the training data set.

We will evaluate the performance of the trained model in terms of 2 perspectives: (1) predictability of missing attributes and (2) representability of the empirical distribution. For the first perspective, we will randomly generate missing patterns on the testing set and measure the prediction accuracy. For the second perspective, we will manually compare the joint distribution of the model and the data on several attribute pairs that would have a strong relationship (eg, age and blood pressure) to ensure that there is no notable discrepancy.

#### Individual Relationships Among the Obtained Multidimensional Data

Normally distributed data will be presented as mean (SD), median (IQR), and number (%), and nonnormally distributed data will be presented as IQR and number (%). The Kolmogorov-Smirnov test will be used to test whether the values of the variables are normally distributed. Analyses of both per-protocol and intention-to-treat groups will be conducted. The areas under the receiver operating characteristic curve (AUCs) and incremental AUCs will be calculated according to the trapezoidal rule from the measurements at each time point during the oral glucose tolerance test. Regression analysis will be used to explore the independent effects of each variable on secondary outcomes. We will examine the relationships among the overall outcomes. Correlations among the overall outcomes will be evaluated using both Pearson correlation coefficient and Spearman rank correlation. In addition, appropriate stratification and partial correlation analyses will be performed in the overall interaction analysis. All statistical analyses of the data will be performed using SPSS software (version 21.0; IBM Corp), SAS (version 9.4; SAS Institute Inc), and R software (R Foundation for Statistical Computing).

## Results

The study was registered at University Hospital Medical Information Network on October 14, 2021. Recruitment was started on October 19, 2021, and ended on February 25, 2022. A total of 997 participants were enrolled in the study. Sample analysis will be performed from February 2022 to March 2023. Data analysis will be conducted in 2023, and the final report will be available in 2024.

## Discussion

### Principal Findings

High-dimensional cross-sectional studies are rarely conducted. We believe that there are several methods to use such data sets. We plan to provide the VHGM that we are developing as a commercial service via an application program interface (API) on the internet. Any application program can send a query to the VHGM, such as *what is the mean and variance of systolic blood pressure of Japanese adults? (marginal probability)*; *what is the probable distribution of blood glucose levels given my age, sex, and lifestyle (excursive, drinking, and smoking habits)? (conditional probability)*; and *what would my estimated cognitive function index be if my age were 10 years older (counterfactual inference)*. We are expecting to discover many innovative health care applications using the VHGM API.

### Strengths and Limitations

Our exploration of individual relationships among the thousands of variables in the data set is expected to reveal previously unknown relationships, which may lead to a new health care hypotheses and further studies, including those aimed at constructing new intervention tests. Thus, this cross-sectional data set will lead to new discoveries in health care research.

Limitations of this protocol include its cross-sectional study design at a point in time, the fact that recruitment (via a website) was restricted to those who could participate in person to perform the survey. In addition, our sampling methods, such as the study’s inclusion criteria and the decision by the responsible physician whether to allow participation, may have introduced a selection bias, making it difficult to generalize our findings to the population. Appropriate weighting can be applied to reduce the impact of selection bias on the estimates. If any modifications to this study design are necessary, we will notify the IRB and submit a study protocol with the necessary changes for IRB review. Thus far, data on preventive medicine and health care have been obtained on an opportunistic and time-consuming basis, using funds as needed. The data obtained from this study have the potential to provide a standardized model for the Japanese population regarding health care and to reveal public health implications. Using a larger sample size would reduce the risk of type II errors.

### Conclusions

The results of this study may provide evidence for the future application of a VHGM based on a joint probability distribution model in clinical health care practice.

## Abbreviations

AGE: advanced glycation end product
APDS: 3-aminopyridyl-N-hydroxysuccinimidyl carbamate
API: application program interface
AUC: area under the receiver operating characteristic curve
FU: follicular unit
HIVAE: heterogeneous incomplete variational autoencoder
HPLC: high-performance liquid chromatography
IL: interleukin
IRB: institutional review board
LC: liquid chromatography
LC-MS/MS: liquid chromatography–tandem mass spectrometry
LC-MS: liquid chromatography–mass spectrometry
MS: mass spectrometry
PCR: polymerase chain reaction
rRNA: ribosomal RNA
SPME-GC-MS: solid-phase microextraction–gas chromatography–mass spectrometry
SSL: skin surface lipid
STROBE: Strengthening the Reporting of Observational Studies in Epidemiology
UMIN: University Hospital Medical Information Network
VHGM: Virtual Human Generative Model

## Data Availability

The data are not publicly available owing to ethical restrictions. Owing to the nature of this research, the participants of this study did not agree to share their data publicly, and thus, supporting data are not available. The statistical properties of the data are approximated in the model and are available via an application program interface.
